# Hypothesis: primary antiangiogenic method proposed to treat early stage breast cancer

**DOI:** 10.1186/1471-2407-9-7

**Published:** 2009-01-08

**Authors:** Michael W Retsky, William JM Hrushesky, Isaac D Gukas

**Affiliations:** 1 Department of Vascular Biology, Children's Hospital and Harvard Medical School, 300 Longwood Avenue, Boston 02115, MA, USA; 2 The University of South Carolina, Dorn VA Medical Center, 6439 Garners Ferry Road (151), Columbia, SC 29209, USA; 3 School of Medicine, Health Policy and Practice, University of East Anglia, Norwich, NR4 7TJ, UK

## Abstract

**Background:**

Women with Down syndrome very rarely develop breast cancer even though they now live to an age when it normally occurs. This may be related to the fact that Down syndrome persons have an additional copy of chromosome 21 where the gene that codes for the antiangiogenic protein Endostatin is located. Can this information lead to a primary antiangiogenic therapy for early stage breast cancer that indefinitely prolongs remission? A key question that arises is when is the initial angiogenic switch thrown in micrometastases? We have conjectured that avascular micrometastases are dormant and relatively stable if undisturbed but that for some patients angiogenesis is precipitated by surgery. We also proposed that angiogenesis of micrometastases very rarely occurs before surgical removal of the primary tumor. If that is so, it seems possible that we could suggest a primary antiangiogenic therapy but the problem then arises that starting a therapy before surgery would interfere with wound healing.

**Results:**

The therapy must be initiated at least one day prior to surgical removal of the primary tumor and kept at a Down syndrome level perhaps indefinitely. That means the drug must have virtually no toxicity and not interfere meaningfully with wound healing. This specifically excludes drugs that significantly inhibit the VEGF pathway since that is important for wound healing and because these agents have some toxicity. Endostatin is apparently non-toxic and does not significantly interfere with wound healing since Down syndrome patients have no abnormal wound healing problems.

**Conclusion:**

We propose a therapy for early stage breast cancer consisting of Endostatin at or above Down syndrome levels starting at least one day before surgery and continuing at that level. This should prevent micrometastatic angiogenesis resulting from surgery or at any time later. Adjuvant chemotherapy or hormone therapy should not be necessary. This can be continued indefinitely since there is no acquired resistance that develops, as happens in most cancer therapies.

## Background

While the mortality rate has been dropping in recent years, breast cancer is diagnosed in 180,000 women and men and kills over 40,000 yearly in the US [1]. When breast cancer is first diagnosed, the patient is given a work-up to determine if there is any evidence of distant metastases. If there is no overt sign of distant metastases, the stage is considered early. If there is evidence of distant metastases at diagnosis or at any time later in the disease process, the stage is called late. Over 90% of new cases of breast cancer are diagnosed at an early stage.

There is determined effort to detect breast cancer at the earliest possible time since outcome after just surgery is more often favorable than it is compared to detection later. For example, women whose primary tumor is 1 cm in size and without axillary lymph nodes involved with cancer can expect 90% probability of cure after only removal of the primary tumor. On the other hand, a patient with 5 cm tumor and 10 lymph nodes with cancer can expect only 10% probability of cure with simple surgical removal of the primary tumor. Patients rarely die from the primary tumor but from later distant relapse of the cancer.

After surgery to remove the primary tumor, adjuvant therapy is often administered to help prevent or delay any possible appearance of distant metastases in the next 15–20 years. It may be in the form of cytotoxic chemotherapy or less toxic hormone therapy. There are well-established means and guidelines to determine which if either or both of these therapies is indicated for any particular patient.

However, treatment for early stage breast cancer too often ultimately fails in that metastatic disease is discovered within 15 or so years after initial diagnosis. Adjuvant chemotherapy improves absolute cure rates by up to 15%. Hormone therapy has approximately the same level of benefit.

Treatment for metastatic disease is still mainly palliative in that long term survival is rare. The median duration of survival after relapse from early stage breast cancer is two years. There is an urgent need for improved treatments for early stage breast cancer that are far more effective in preventing relapses for long periods of time – hopefully until the person dies of another disease or old age. Based on the experience over the past few decades, we are more likely to make an impact by learning how to more effectively prolong remission in early stage breast cancer than we are in learning how to eradicate a tumor that is macroscopic in size.

Based on our computer simulations of clinical data, when a person is diagnosed with early stage breast cancer, it is rare that any sites of metastatic disease deposits have achieved angiogenesis. That is, at the time of detection, other than the primary tumor, there are usually only disseminated distant dormant single cancer cells and distant dormant avascular deposits present [2,3]. In human breast cancer, single dormant cells have been proven but (more difficult to observe) dormant avascular micrometastases have not yet been visualized [4].

A surprising finding from our analysis was that the surgery to remove the primary tumor apparently often kick-starts growth of the dormant cells and avascular micrometastases. Most relapses occur within the first 5 years after surgery and are mainly events that are triggered into growth from surgery. We have suggested that one of the side effects of surgery is to stimulate division of dormant single malignant cells and stimulate angiogenesis of dormant micrometastases. The latter is most apparent for the premenopausal node-positive population. According to these reports, 20% of premenopausal node-positive patients undergo surgery-induced angiogenesis and over half of all relapses in breast cancer are accelerated by surgery [5]. There is no direct evidence that this quantitative prediction is correct but there is strong indirect evidence mainly from mammography data that it is valid and numerically accurate [6-9]. For example, our analysis predicted that in trials of early detection conducted before the routine use of adjuvant therapy, there would be 0.1 excess deaths per 1000 screened premenopausal women in the second or third year after the start of screening. This is consistent with data based on 1.6 million person-years of follow-up from mammography trials conducted in different decades and in different countries. Surgery-induced angiogenesis for premenopausal patients with positive nodes is similar to the O'Reilly et al Lewis lung study [10].

These surgery-induced effects reduce the benefit of early detection. Most persons derive benefit from early detection since they will be diagnosed with less extensive disease but paradoxically other persons will relapse and die earlier as an unfortunate consequence of early detection. This counterintuitive effect is most apparent in young women.

Our more recent work suggests that this can partially explain the excess breast cancer mortality of African-Americans, since the average age of diagnosis of African-Americans is 46 years compared to 57 years for European-Americans. This excess first appeared in the 1970s when mammography began [11,12].

Perhaps not coincidentally, adjuvant chemotherapy works best by far for premenopausal patients who are node-positive. According to our theories, the reason for this is that sudden metastatic tumor growth just after surgery produces a chemosensitive window just at the time when adjuvant therapy was empirically found to be most effective. The implication is that surgery produces a disruption and acceleration of disease and then adjuvant chemotherapy is used to address the effects of the disruption [13].

In 2005 we analyzed data from an adjuvant hormone therapy trial comparing Tamoxifen and Arimidex [14]. As we reported, hormone therapy mainly suppresses relapses that would have occurred in the first 5 years after surgery. Other information along those lines is that Tamoxifen, the most frequently used hormone therapy drug, is useful in the first five years after surgery. After that time, Tamoxifen has not been demonstrated to be of value. One way of interpreting these data is that adjuvant hormone therapy, like adjuvant chemotherapy, functions to counteract surgery-induced growth of micrometastatic disease.

Working on the assumption that this is all true, we have proposed that antiangiogenic drugs given when disease is still microscopic would be very helpful but that this treatment should be initiated before surgery since it is far more difficult to reverse angiogenesis after it is established than it is to prevent it from happening before it occurs [15]. Naumov et al suggest that if surgery induces angiogenesis of dormant micrometastases, antiangiogenic, anti-inflammatory or other growth inhibiting drugs administered before and/or after surgery might reduce relapse within 18 months of surgery [16].

The undisturbed half-life of avascular micrometastases in breast cancer is 2 years and the undisturbed half-life of single dormant cells is 1 year [2]. This suggests that the avascular dormant state is the more stable of the two dormant states. Efforts to prolong the natural tendency of dormancy of disease in these early states, especially the pre-angiogenic state, could be pursued as one method of cancer control that would surely reduce cancer mortality.

Administering an antiangiogenic drug around the time of surgery presents a conundrum since wound healing after surgery highly depends on angiogenesis to remodel and rebuild tissue [17]. So it would appear that starting an antiangiogenic therapy before surgery and continuing it to prevent micrometastases from escaping dormancy would interfere with wound healing after primary tumor removal. This seems to preclude using an antiangiogenic therapy around the time of surgery. It would be very important if a way could be found to treat early stage breast cancer with an effective antiangiogenic drug for an indefinite time starting before primary surgery but yet not interfere with wound healing resulting from the surgery. Is there any possible way around this apparent impasse?

## Presentation of hypothesis

### Down syndrome and the possible role of trisomy 21 in antiangiogenesis

Endostatin is the C-terminus fragment of collagen XVIII (blood clotting function) and is a very robust inhibitor of angiogenesis. The mechanism is thought to be an inhibition of endothelial cell migration and also apoptosis. It is endogenous and non-toxic. In fact, unique in the history of the FDA testing program, it has never been shown to exhibit toxicity at any level at any concentration. It has, in fact, been suggested that Endostatin be given to healthy persons to diminish cancer as a public health concern [18-20].

In support of that argument, Folkman and Kalluri [18] have pointed out that persons with Down syndrome (DS) rarely have breast cancer (10 – 25 fold less than age-matched normals) and that they also have an elevated level of Endostatin [20-22]. This is correlated to the genetic defect in that DS persons have between two and three copies of chromosome 21 which harbors the Endostatin gene.

According to Greene et al [23] there are at least 283 protein encoding genes in chromosome 21, which corresponds to approximately 1% of the human DNA. This chromosomal defect responsible for the DS phenotype also codes for collagen XVIII so, on average, DS persons have more Endostatin than those with normal chromosome 21. The ratio is approximately 1.8 according to Zorick et al [20] and 1.48 according to Greene et al [23].

One possible explanation for the lack of breast cancer in DS is the high level of Endostatin resulting from trisomy 21. As another explanation, since collagen XVIII is a major component of the basal membrane of tumor-associated blood vessels, perhaps its uncontrolled expression is responsible for a relative defect in tumor angiogenesis in DS.

DS often have congenital heart disease that is repairable with surgery so there are data on wound healing. Lange et al reported on results of surgery to repair complete atrioventricular septal defect in 476 patients, 71.6% of who were DS and the remainder normal [24]. There was 30 day mortality of 4.9% in the DS and 5.6% in those with normal chromosomes. There was more frequent pulmonary hypertension among DS but there was no difference in operational strategy or timing of repair. It was concluded that the presence of DS was not a risk factor for surgical repair of complete atrioventricular septal defect. While this might suggest Endostatin has no antiangiogenic activity, we think a better explanation is that Endostatin at the DS level does not adversely affect wound healing.

Endostatin was patented December 29, 1998. This drug has not lived up to the very high initial expectations of benefit. Phase I and Phase II clinical trials were conducted following the highest standard of clinical trial methodology and translational research [25-28] however these trials failed to show any significant anti-tumor activity or abnormalities of wound healing or in tumor angiogenesis. This is in contrast to the positive results from trials of other antiangiogenic compounds Bevacizumab and Sunitinib. However, Endostatin has occasionally and dramatically stabilized disease in a few otherwise hopeless cases. In addition, the original Boehm et al [29] results of Endostatin in animal models have not been reproduced. Overall, it is fair to say that previous efforts to induce tumor dormancy with continuous high levels of Endostatin have produced mixed results [30-32].

From our perspective, Endostatin would be more effective to prevent angiogenesis before it happens in breast cancer, a disease with an apparent strong tendency to remain dormant at least prior to primary tumor removal. The previous use of continuous high levels of Endostatin did not work well, perhaps because it was started too late in the disease life cycle (after angiogenesis had already happened). We think that studies using early stage breast cancer may be more likely to be beneficial.

A molecule very similar to Endostatin called Endostar has been manufactured in significant quantities by a company in China. This drug has been tested in the Folkman lab and found to be twice as effective as early samples of Endostatin. [Recent developments have improved Endostatin half-life from hours to weeks [33].] Endostar is currently used in China for late stage lung cancer patients but is not currently approved for use in the US. No clinical data have been published yet in a peer review journal.

According to Xu et al, Endostatin can maintain tumors in a state of dormancy although they report that the half-life is short so Endostatin is best utilized with prolonged delivery using mini-osmotic pumps or cell encapsulations systems [34]. They also report results are best when the drug is administered as early as possible and no evidence of drug resistance has been seen.

It has been suggested that 1.6 or 1.7 fold increase of Endostatin relative to average normal level will prevent angiogenesis. Zorick et al, however, have suggested that only 30% more Endostatin than normal will effectively prevent angiogenesis [20]. There might be no acquired resistance to this therapy judging by the DS data. That is important since it is widely accepted that conventional chemotherapy and hormone therapy drugs eventually cease to be effective due to acquired drug resistance.

Zorick et al reported levels of Endostatin in normals and DS subjects. Levels for normal controls were 20.3 +/- 11.5 ng/ml with range of 4 to 40. For Down syndrome subjects, the levels were 38.6 +/- 20.1 ng/ml with range of 6 to 76. The sensitivity of the test kit was 2 ng/ml with typical intra- and inter-assay variances of 10% or less.

Bevacizumab, a monoclonal antibody against VEGF, has been available for several years and has made a major impact especially in late stage colon cancer. It has only been beneficial when combined with a conventional cytotoxic chemotherapy drug. As a monotherapy it has not demonstrated value. No long term cures have been claimed from use of Bevacizumab although the duration of survival with metastatic colon cancer is markedly improved. VEGF is a very important angiogenic pathway in cancer. However there are many angiogenesis pathways so shutting off one pathway may not prevent angiogenesis from progressing via another pathway. Bevacizumab displays some dose limiting toxicity mainly hypertension.

## Hypothesis – primary antiangiogenic therapy proposed

Data presented in a paper by Wu et al in 2003 suggest the post cancer resection dynamics of VEGF and Endostatin in the wound fluid and in plasma are quite distinct [35]. As they report, mastectomies for a number of breast cancer patients and female-to-male sex change cases were used to measure angiogenesis inhibitors and promoters before and after surgery. Endostatin and VEFG were measured in plasma and wound fluid days 1 and 4 post surgery plus Endostatin baseline was measured prior to surgery. VEGF increased very significantly (9-fold) in wound fluid but not in plasma. Endostatin decreased significantly and temporarily by 20 – 30% in plasma but did not change in wound fluid. The Endostatin decrease appeared at day surgery+1 but then almost returned to presurgery levels by surgery+4.

VEGF but not Endostatin is involved in wound healing according to Wu et al data. Wu et al data on wound fluid and plasma suggest that there are at least two important and distinct pathways for angiogenesis in early stage breast cancer. One pathway is for wound healing involving temporarily highly upregulated VEGF in the local wound area and another pathway is for systemic stimulation of tumor angiogenesis by temporarily down-regulating Endostatin. This interpretation of their data apparently was not noticed by Wu et al.

This temporary dip in naturally occurring angiogenesis inhibitors such as Endostatin is what produces the surgery-induced angiogenesis. Sund et al mentions Thrombospondin and Tumstatin as endogenous suppressors of angiogenesis in addition to Endostatin [32]. This suggests that if the level of endogenous inhibitors such as Endostatin, Angiostatin, Tumstatin, Thrombospondin or any antiangiogenic acting protein from chromosome 21 such as NC1 in plasma could be kept high at least for those few critical days, it might prevent distant angiogenesis while not interfering with wound healing. According to Errera et al, Endostatin is a fragment of NC1 [36]. According to Roh et al, Celecoxib and Indomethacin are also effective in preventing wound healing associated tumor growth so those drugs may be also considered in the list [37]. Celecoxib was most beneficial when started 1 day before surgery in animal models. There have been some suggestions that Celecoxib may have some long term toxicity. The immunostimulant Taurolidine also can prevent surgery induced tumor growth according to Da Costa et al so that drug may also be a candidate although the authors suggest this may be a result of cells released following surgery [38].

**Figure 1:**
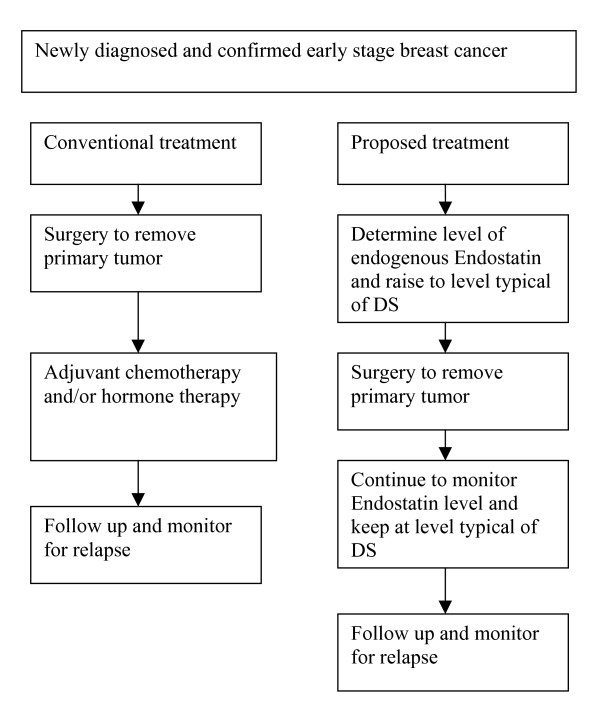
**Comparison of conventional treatment and proposed primary antiangiogenic therapy for early stage breast cancer**.

We hypothesize that subtle interruption of the initiation of angiogenesis within micrometastatic tumor deposits by increasing endogenous Endostatin will prevent disease progression as long as the level is retained but it must begin before surgical resection. Taking a clue from the DS situation where 1.3 to 1.8 times the level of Endostatin in serum reportedly would prevent most solid tumors over the life of the subject, an approximate value of Endostatin to retain is at least 1.3 – 1.8 times the serum level in normal subjects. The amount of Endostatin to be added will thus depend on the particular individual. Some may not need any additional Endostatin beyond the first critical few days post surgery. In addition, based on the Wu et al data, the effect of surgery-induced angiogenesis is not tied to removing any particular cancer but is a consequence of general surgery. This strategy, shown in fig. 1, will apply to any cancer patient, especially early stage, who has any surgery.

While unlikely, long term use of Endostatin at the DS level might have side effects. For example, since angiogenesis is essential for follicle formation, it may induce castration in premenopausal women. If that happened, improved survival for premenopausal women would result. Of course that is not a bad result but it would complicate outcome analysis. Such events need to be monitored in a trial.

## Implications of the hypothesis

We have proposed a new method of therapy for early stage breast cancer patients. It is designed to prevent angiogenesis and should keep all metastatic deposits microscopic for as long as the therapy is continued without limit. No drug resistance or toxicity is expected.

This therapy we suggest may or may not eradicate the micrometastases even if given over a long time but it could prevent growth beyond a mm or so indefinitely. The advantages are important. First, adjuvant chemotherapy and adjuvant hormone therapy might prove to be unnecessary since they seemingly serve to counteract surgery-induced cell division and angiogenesis. Second, with relatively long 2 year half-life, avascular dormancy is a naturally very stable situation and would be far easier to maintain long term with a low toxicity antiangiogenesis inhibitor in comparison to eradicating metastases with chemotherapy, radiation, surgery or antiangiogenic therapy after they start to grow. Third, wound healing would be unimpaired while an anti-VEGF drug such as Avastin would very probably interfere with wound healing. Fourth, this therapy could be continued for ensuing years at appropriate elevated levels and may prevent future relapses for all early stage breast cancer patients. Fifth, this therapy takes full advantage of early detection and there will be no paradoxical disadvantage to anyone diagnosed early including premenopausal women. Sixth and most important, mortality from breast cancer will be reduced. Seventh, there should be no racial differences in outcome. Eighth, this would be an ideal therapy for developing countries where there is a minimum of health care funds and supportive infrastructure such as medical specialists, imaging equipment and well-equipped pathology labs.

Breast cancer is the most obvious, but this idea could be applied to other cancers as well. Lung cancer, melanoma, ovarian, cervical, prostate, and osteosarcoma [39] come to mind. This therapy should work without need for adjuvant chemotherapy, radiation, Herceptin for HER2 positive patients, or adjuvant hormone therapy. Perhaps even removal of the primary tumor may be unnecessary for some patients. The money saved by avoiding tests and not needing those modalities would help offset the costs of using Endostatin. While it is likely unreasonable to give Endostatin to every healthy person as a preventative, it is far more reasonable and economical to give it to every cancer patient especially if this therapy prevents relapse since that is when most of cancer care expenses occur.

### Testing the hypothesis

Before we discuss a clinical test of this hypothesis, there are a few other ways of testing that we may suggest. The Wu et al study as well as our interpretation of their data need verification. Perhaps autopsy studies of DS women could look for the presence of ductal carcinoma in situ (DCIS). If there is significant DCIS yet no breast cancer, that might indicate carcinogenesis occurs normally in DS but does not develop into breast cancer and perhaps inhibition of angiogenesis is the reason. Can avascular dormant micrometastases be observed and documented in human breast cancer? Another test is with animal models. Does a pre-established DS level of Endostatin prevent cancer from developing after inoculation with cancer cells in some system that has some predisposition to dormancy such as a breast model [40]?

The easiest way this idea could be tested in clinical situations would be to start with 50 or more consecutive high risk, willing, Stage II breast cancer patients having 4 or more positive lymph nodes. These would be randomized half into controls given best conventional therapy and half test subjects given best conventional therapy plus Endostatin at 1.8 times normal levels starting a few days before surgery. This therapy would continue for 2–3 years or longer, depending on results. Within a few years there should be a significant difference in outcome if the idea is correct. Afterwards, trials could be done to determine if adjuvant therapy is needed at all.

## Competing interests

MR has applied for a US patent for a primary antiangiogenic therapy for early stage cancer. Other than that, authors declare that they have no competing interests.

## Authors' contributions

MR did the computer simulation, conceived of this study and drafted the manuscript. WJH participated in explanation of how surgery could induce angiogenesis, how that may explain breast cancer data, and participated in writing the manuscript. IDG participated in the explanation of the racial differences in outcome that may be explained by surgery induced angiogenesis for premenopausal women and participated in writing the manuscript. All authors read and approved the final manuscript. Funding agencies had no role in study design, interpretation of data, in the writing of the manuscript or in the decision to submit it for publication.

## Pre-publication history

The pre-publication history for this paper can be accessed here:

http://www.biomedcentral.com/1471-2407/9/7/prepub
